# Supplementary material for: Genome-Wide Identification and Functional Analysis of C2H2 Zinc Finger Transcription Factor Genes in the Intertidal Macroalga Pyropia haitanensis
Source: Int J Mol Sci. 2025 Apr 24;26(9):4042. doi: 10.3390/ijms26094042 (PMC12071319; doi:10.3390/ijms26094042)
Supplement: Supplementary file 1 [file ijms-26-04042-s001.zip › supplementary legend.pdf]

Supplementary Materials.

Supplementary Figure S1. Bioinformatics analysis of *PhC2H2.94*. A. Predicted secondary structure of *PhC2H2.94*. B. Phylogenetic tree for *PhC2H2.94* constructed according to the neighbor-joining method. C. Predicted tertiary structure of *PhC2H2.94*. D. Multiple sequence alignment and conserved domains in *PhC2H2.94*.

Supplementary Figure S2. *PhC2H2.94* expression in transgenic *C. reinhardtii* and assessment of stress resistance. A. Agarose gel electrophoresis-based verification of transformation; WT, wild-type *C. reinhardtii*; 1–3, transgenic *C. reinhardtii*. The target fragment was amplified for transgenic *C. reinhardtii*, but not for wild-type *C. reinhardtii*. B. Segmented cloning comparison results. C. Transgenic *C. reinhardtii* and wild-type *C. reinhardtii* exposed to high-temperature stress (32 °C). D. $OD_{750\ nm}$ of *C. reinhardtii* under high-temperature stress conditions. E. *PhC2H2.94* expression pattern in transgenic *C. reinhardtii* under high-temperature stress (32 °C) conditions. F. The expression levels of antioxidant enzymes and heat shock protein genes of wild-type (WT) and transgenic (T2) *C. rheinalis* under high temperature stress.

Supplementary Table S1. TF family statistics. TF family counted the types and numbers of transcription factors in representative red algae, *O. sativa* and *A. thaliana*, and counted the number of *C2H2* transcription factors in more representative species.

Supplementary Table S2. primers. BSA counted the results of QTLs and screened C2H2-type domain-containing protein. *C2H2* is the gene-related information further found in the genome. Primer is the primer used in this experiment.

Supplementary Table S3. Cis-acting, physicochemical, properties. The cis-acting element is a cis-acting element of the *PhC2H2* gene family obtained by genome-wide analysis. Property is the predicted physical and chemical properties of the *PhC2H2* gene family. Transcriptome is the expression profile of the *PhC2H2* gene family at different developmental stages and under different stress treatments.

Supplementary Table S4. Gene structure and transposons. Intron counted the number of introns and exons of the *C2H2* gene family in red algae. TE counted the number and type of TE insertions in three *Pyropia*/*Porphyra*. PH, PY, PU represented TE insertions corresponding to *P. haitanensis*, *P. yezoensis*, *P. umbilicalis*.
