# Supplementary figures and images for: Genome-Wide Identification and Functional Analysis of C2H2 Zinc Finger Transcription Factor Genes in the Intertidal Macroalga Pyropia haitanensis
Source: Int J Mol Sci. 2025 Apr 24;26(9):4042. doi: 10.3390/ijms26094042 (PMC12071319; doi:10.3390/ijms26094042)

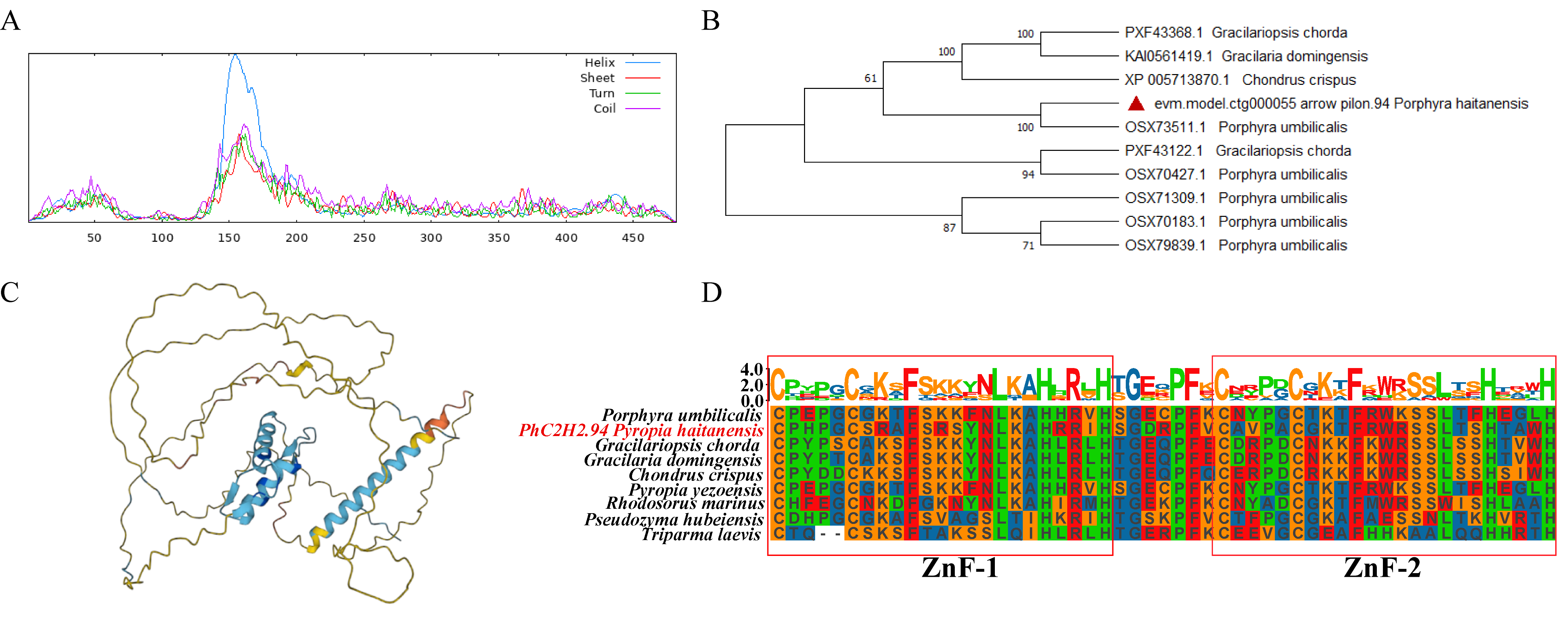

Supplement: Supplementary file 1 [file ijms-26-04042-s001.zip › Figure S1.tif]
